# *Melaleuca alternifolia* Concentrate Inhibits *in Vitro* Entry of Influenza Virus into Host Cells

**DOI:** 10.3390/molecules18089550

**Published:** 2013-08-09

**Authors:** Xinghua Li, Songwei Duan, Cordia Chu, Jun Xu, Gucheng Zeng, Alfred King-Yin Lam, Junmei Zhou, Yue Yin, Danyun Fang, Maxwell John Reynolds, Huaiyu Gu, Lifang Jiang

**Affiliations:** 1 Key Laboratory of Tropical Disease Control of Ministry of Education, Department of Microbiology, Zhongshan School of Medicine, Sun Yat-sen University, Guangzhou 510080, China; E-Mails: lixh1719@hotmail.com (X.L.); zenggch@sysu.edu.cn (G.Z.); junmeizh@126.com (J.Z.); lycwr@hotmail.com (Y.Y.); danyunf@126.com (D.F.); 2 Department of Anatomy, Zhongshan School of Medicine, Sun Yat-sen University, Guangzhou 510080, China; E-Mail: duansw@mail2.sysu.edu.cn; 3 Center for Environment and Population Health, Griffith University, Queensland 4111, Australia; E-Mails: c.chu@griffith.edu.au (C.C.); professor-reynolds@neumedix-biotech.com (M.R.); 4 Research Center for Drug Discovery and Institute of Human Virology, School of Pharmaceutical Sciences, Sun Yat-sen University, Guangzhou 510006, China; E-Mail: xujun9@mail.sysu.edu.cn; 5 Cancer Molecular Pathology, Griffith Health Institute, Griffith University, Gold Coast, QLD 4222, Australia; E-Mail: a.lam@griffith.edu.au

**Keywords:** *Melaleuca alternifolia* Concentrate (MAC), influenza virus, haemagglutinin, terpinen-4-ol, molecular docking, molecular dynamics

## Abstract

Influenza virus causes high morbidity among the infected population annually and occasionally the spread of pandemics. *Melaleuca alternifolia* Concentrate (MAC) is an essential oil derived from a native Australian tea tree. Our aim was to investigate whether MAC has any *in vitro* inhibitory effect on influenza virus infection and what mechanism does the MAC use to fight the virus infection. In this study, the antiviral activity of MAC was examined by its inhibition of cytopathic effects. *In silico* prediction was performed to evaluate the interaction between MAC and the viral haemagglutinin. We found that when the influenza virus was incubated with 0.010% MAC for one hour, no cytopathic effect on MDCK cells was found after the virus infection and no immunofluorescence signal was detected in the host cells. Electron microscopy showed that the virus treated with MAC retained its structural integrity. By computational simulations, we found that terpinen-4-ol, which is the major bioactive component of MAC, could combine with the membrane fusion site of haemagglutinin. Thus, we proved that MAC could prevent influenza virus from entering the host cells by disturbing the normal viral membrane fusion procedure.

## 1. Introduction

Influenza is an infectious disease caused by the influenza virus which is a RNA virus of the family Orthomyxoviridae. Influenza spreads around the world in seasonal epidemics, with an estimated three to five million cases of severe illness and 250,000 to 500,000 deaths per annum [1]. Four major influenza pandemics occurred in the 20th century that caused more than 20–50 million deaths, and influenza virus infection remains one of the leading causes of mortality [2,3]. A new H1N1 influenza A virus, also called the 2009 H1N1 pandemic influenza virus (2009 H1N1 virus), had spread throughout the world and caused a serious influenza pandemic in 2009 [4,5]. Over 17,000 reported deaths have been caused by 2009 H1N1 virus infection since its identification in Mexico in April 2009, so drugs and vaccines against 2009 H1N1 virus infection are urgently needed [6]. However, 2009 H1N1 virus, like many other influenza virus stains, has developed resistance to commercially available anti-influenza drugs. Currently the neuraminidase (NA) inhibitor oseltamivir, which can interfere with the enzymatic activity of the NA of the influenza virus, is mainly used for the treatment of influenza patients, but the 2009 H1N1 virus has been reported to be resistant to it [7,8]. It has been recently reported that over 160 sporadic viral isolates of 2009 H1N1 virus show resistance to oseltamivir due to the NA H275Y genotype mutation [8,9]. On the other hand, though the vaccines against 2009 H1N1 virus infection have been developed and used in clinical practice, the safety of theses vaccines remains one of the major public concerns in most of countries [10,11,12,13], as deaths and serious side effects of vaccines against 2009 H1N1 virus have been reported [14].

The haemagglutinin (HA) on the surface of influenza virus particles is a major viral membrane glycoprotein molecule, which is synthesized in the infected cell as a single polypeptide chain precursor (HA0) with a length of approximately 560 amino acid residues and subsequently cleaved by an endoprotease into two subunits called HA1 and HA2 and then be covalently attached by the disulfide bond [15,16]. The crystallographic structure of the HA shows a long tightly intertwined fibrous stem domain at its membrane-proximal base, a globular head which contains the sialic acid receptor binding site (RBS) and five antigenic sites surrounding the RBS [17]. The mature HA on the viral surface is a trimeric rod-shaped molecule with the carboxy terminus inserted into the viral membrane and the hydrophilic end forming the spike of the viral surface [18,19,20]. Although the amino acid sequence identity of different virus strains can be less than 50%, the structure and functions of these HAs are highly conserved [16]. The major functions of the HA are as the receptor-binding ligand, leading to endocytosis of the virus into the host cell and subsequent membrane-fusion events in the infected cells [16,21]. Influenza virus initiates infection by binding to sialic acids on the surface of target cells. After endocytosis, the endosome acquires a lower pH value, mainly because of the activity of the Vacuolar-type H+-ATPase (V-ATPase) [22]. In the acid environment of the endosome, the HA molecule is cleaved into HA1 and HA2 subunits and then undergoes a conformational change which resulting in the exposure of the fusion peptide at the *N*-terminus of the HA2 subunit [23,24]. The fusion peptides insert into the endosomal membrane, while the transmembrane domains remain anchored in the viral membrane. Finally, the fusion peptide brings the endosomal membrane and the viral membrane into juxtaposition, leading to fusion. Subsequently, a pore is opened up by this structural change of more than one haemagglutinin molecule and then the contents of the virion are released into the cytoplasm of the cell. This completes the uncoating process [25]. Because of the conformational change of viral HA protein is indispensable for the membrane fusion process between influenza virus and the endosome of the host cell, this makes it a new target for anti-influenza virus drug development. Recently, some small compounds acting as HA conformational change inhibitors have been reported [26,27].

Herbal extracts have been reported to have an important role in controlling virus infections by serving as immuno-modulators during influenza virus infection [28] or blocking the interaction of virus with target cells or having virucidal activity through direct interaction with the virus [29,30]. Most importantly, accumulating evidence has suggested that treatment of herbal extracts might be able to reduce the risk of drug-resistant virus emergence [31]. *Melaleuca alternifolia* Concentrate (MAC), which is an essential oil derived from the leaves or terminal branches of the native Australian tea tree, *Melaleuca alternifolia*, is a heterogeneous mixture of approximately 100 chemically defined components that mainly contains terpinen-4-ol (56%–58%), γ-terpinene (20.65%), and α-terpinene (9.8%) [32]. The ability of MAC to induce anti-inflammatory effect [33,34] and inhibit infection of various microbial species, such as bacteria [35,36], viruses [37,38,39] and fungi [40,41] makes it a promising candidate for development of therapeutics against 2009 H1N1 virus infection.

The purpose of this study was to determine the antiviral effect against 2009 H1N1 virus using an *in vitro* test of cytopathic effect (CPE) inhibition of MAC. As previously described, terpinen-4-ol was the main component of MAC, so here we also assessed the feasibility and sensitivity of interaction of terpinen-4-ol with the viral haemagglutinin protein through *in silico* prediction to confirm the drug target and the characterization of the protein changes after treatment with MAC.

## 2. Results and Discussion

### 2.1. Cytotoxic Test of MAC

**Figure 1:**
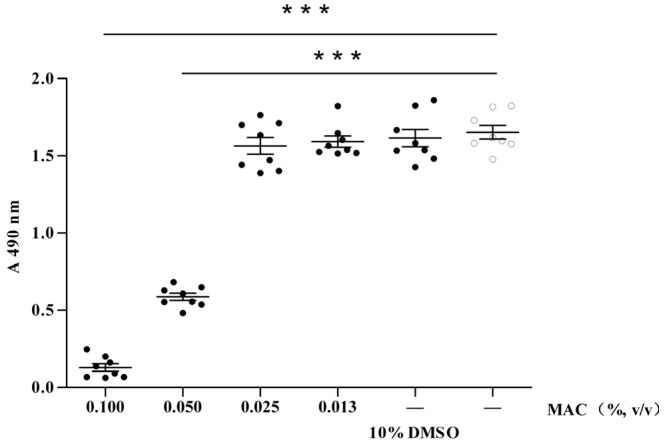
Effects of MAC on the MDCK cell viability.

As an initial step to determine the anti-virus effect of MAC, we first need to determine whether MAC has any effect on cellular viability. To address this, MDCK cells were co-cultured with MAC at various concentrations for about 72 h. The cellular viability of MAC was determined by a MTT assay, which is a colorimetric assay for assessing the viability of cells. Although MAC at concentration higher than 0.050% could induce significant cellular death, it did not have any cytotoxic effect on MDCK cells when the concentration was lower than 0.025%. In addition, 10% DMSO/DMEM control was set up because there was DMSO in the MAC solution. Interestingly, the absorbance value of the cell incubated in 10% DMSO/DMEM was similar to the cell control (Figure 1). This observation also indicated that the cell death was produced by MAC at a high concentrations but not DMSO, because of the concentration of DMSO in the MAC working solution is far lower than 10%. These data suggest that MAC at proper concentrations does not have any cytotoxicity, and since MAC at concentrations lower than 0.025% did not have any cytotoxic effect on MDCK cells, we choose a concentration of 0.020% as a maximum study concentration to further determine the anti-viral effects of the MAC.

MACs of different concentration were applied to the MDCK cell monolayer, the 10% DMSO/DMEM control and the cell control were set up. After 72 h incubation at 37 °C, 5% CO_2_, the viability of MDCK cells were determined by a standard MTT assay protocol, as described in details in Experimental section. The data were presented as means ± S.D. ***: *p* < 0.001.

### 2.2. Anti-Viral Effect Assay of MAC *in Vitro*

**Figure 2:**
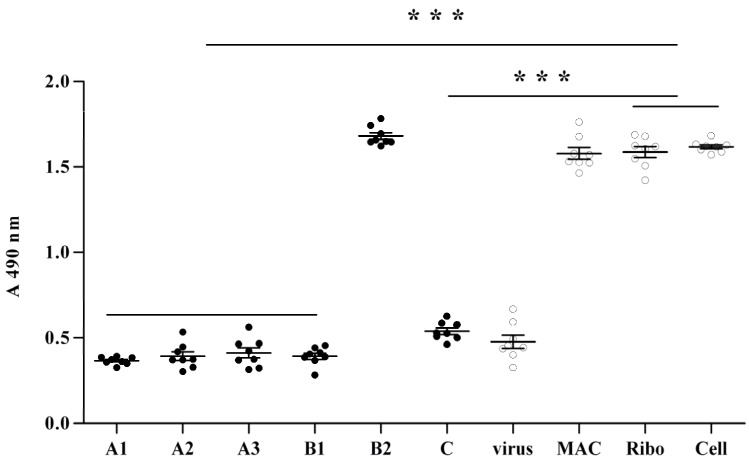
Protection efficacy by MAC against 2009 H1N1 virus infection to MDCK cell.

To determine the anti-viral effect of MAC, we first asked whether MAC could confer protection capability against influenza virus to the cells. To answer this question, MDCK cells were first treated with 0.020% MAC for 1, 2, and 4 h, respectively. MAC was then removed by careful sterile PBS wash. The MDCK cell monolayer was then inoculated with 2009 H1N1 virus in 100 TCID50 per well for 1 h and then the liquid was removed by sterile PBS wash and instead of the maintain media containing TPCK-trypsin 1μg/ml. The viability of MDCK cells were then determined by MTT method, when level 2‒3 CPE was observed in the virus control and the cell control showed no CPE (about 48–72 h). As shown in Figure 2, no significant increase of cellular viability of MDCK cells was observed when MDCK cells were pretreated with MAC for one hour, two hours, and four hours (A1, A2 and A3) respectively, compared with the virus control and significant lower than the cell control and the ribavirin (ribo) control. It was worth noting that, the cell survival under A1, A2 and A3 condition remained the same. In other words, there was no tendency showing the cell survival was increased according to prolonging the time of treatment with MAC. These data, therefore, indicated that pretreatment of MDCK cells with MAC could not confer any cellular viability protection.

Because pretreatment with MAC could not make MDCK cell produce any changes for protecting against influenza virus infection, we then examined whether treatment of the virus but not MDCK cells with MAC could confer any cellular viability protection. To determine this, 2009 H1N1 virus were first treated with MAC at a concentration of 0.010% for 0.5 and 1 h, respectively. The mixtures were added to a MDCK cells monolayer and then washed away after initial 1 h incubation and replaced with maintenance media containing TPCK-trypsin 1 μg/mL. The cellular viability was tested as mentioned above. As shown in Figure 2 (B1 and B2), although the infectivity of the influenza virus treated with MAC for 0.5 h still remained, the virus treated with MAC for 1 h presented poor infection of the host cells. Therefore, these data indicated that the influenza virus treated with MAC would dramatically lose its infective ability towards the host cells.

A: 0.020% MAC was applied to the MDCK cell monolayer for 1 h (**A1**), 2 h (**A2**) and 4 h (**A3**), respectively. MAC in each MDCK cell culture was then removed by extensive sterile PBS wash and then 2009 H1N1 virus in 100 TCID50 were infected for 1 h and then instead of the maintenance media. **B**: MAC was diluted into 0.010% with the final concentration of 10^3^ TCID 50/mL 2009 H1N1 virus suspension for incubation of 0.5 h and 1 h (**B1** and **B2**), respectively. The mixtures were added to MDCK cells monolayer for 1 h and then replaced by maintenance media. **C**: 100 TCID50 influenza viruses were inoculated to a MDCK cell monolayer for 1 h. The supernatant was then replaced by 0.020% MAC/DMEM. Virus, MAC, ribo and cell represent 2009 H1N1 influenza virus control, 0.020% MAC control, 0.010% ribavirin anti the influenza virus control and the MDCK cell control, respectively. The viability of MDCK cells were then determined by MTT method when over level 2 CPE was observed in the virus control and the cell control showed no CPE (about 48 h). The data were presented as mean ± S.D. ***: *p* < 0.001.

Subsequently, the effect of MAC on the influenza virus that has entered into MDCK cells was investigated. The influenza virus was inoculated to a MDCK cell monolayer with 100 TCID50 per well for 1 h to make the virus enter the host cells. The supernatant was then removed by sterile PBS wash and replaced by 0.020% MAC/DMEM containing TPCK-trypsin 1 μg/mL. The cellular viability was tested as mentioned in the Experimental. The results are shown as part C of Figure 2. MAC could not induce any appreciable increase of the cellular viability of MDCK cell compared with the virus control. This indicated that MAC could not prevent influenza virus replication and biosynthesis in the host cell. The new generation of virus produced in the host cell could complete the life cycle and export from the host cell.

**Figure 3:**
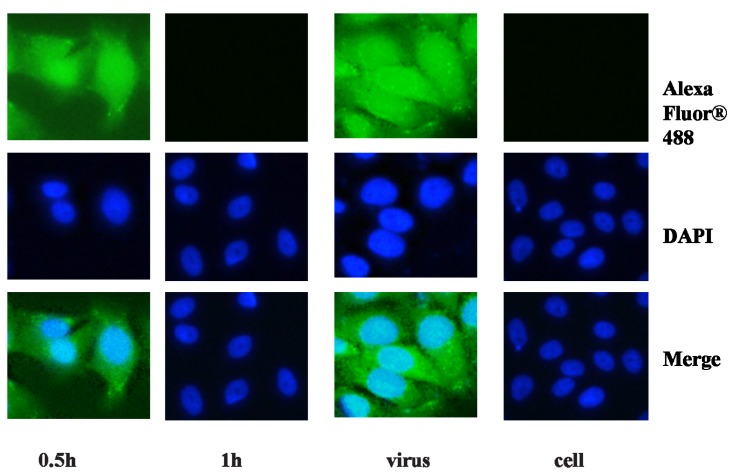
Treatment with MAC prevents the influenza virus entering the host cell.

**Figure 4:**
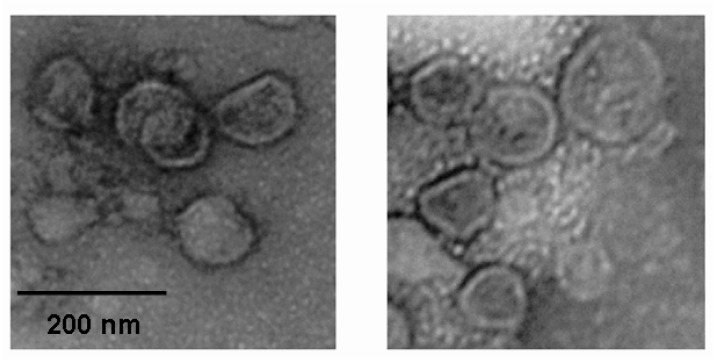
The role of MAC on destroying the structural integrity of the virion.

To observe the proliferation of influenza virus in the host cell intuitively, an immunofluorescence assay was performed. The influenza viruses were treated with MAC to a final concentration of 0.010% for 0.5 and 1 h, respectively and a virus control and a cell control were set up. The primary antibody and the fluorochrome labeled secondary antibody produced cytoplasm staining patterns in MDCK cells infected by the influenza virus treated with MAC for 0.5 h and the virus without treatment, whereas only robust nuclear staining was detected by DAPI in MDCK cells infected by the influenza virus treated with MAC for 1 h and the cell control (Figure 3). In addition, integrity of the virus particle after incubation with MAC was visualized by electron microscopy. No matter whether the influenza virus was treated with MAC or not, numerous whole virus particles could easily be visualized in the images. No changes in the general structure of the virion could be observed (Figure 4). This result demonstrated that MAC could not lyse the virion.

0.010% MAC in the virus suspension incubation for 0.5 h (0.5 h) and 1 h (1 h), the influenza virus untreated (virus) were inoculated respectively to a MDCK cell monolayer in a 96 well plate for 5 h, at the same time a cell control (cell) was set up. The virus was stained intracellularly with influenza A M1 antibody and Alexa Fluor^®^ 488 Goat Anti-Mouse IgG. Double immunofluorescence staining graphs indicated the location of the virus was in the cytoplasm. Note that in the left second line, which showed the virus treated with MAC for 1 h, there was no green immunofluorescence. This served as negative control and in contrast to the virus control showing green immunofluorescence.

The influenza virus was treated with MAC of 0.010% final concentration for 1 h (left) and the virus control (right) was set up. Numerous intact virions could easily be visualized with close density of virions in both groups. The images shown here are representative views of 10 replicates.

### 2.3. Molecular Modeling and Molecular Dynamics Simulation Studies

Given the result that MAC could inhibit 2009 H1N1 virus infection when the MAC was applied before the virus entered MDCK cells, but could not prevent replication and biosynthesis of the virus in the host cell, MAC appears to inhibit entry of influenza virus into the host cell. This involves two key steps: the first is virus attachment to the cell-surface via the receptor site on the HA protein and then internalization within endosomes; the next step involves fusion between the viral envelope and endosomal membrane, mediated by the conformational change in the HA protein, triggering uncoating. Viral nucleocapsids are then released into the cellular cytoplasm for transcription and translation. Since the two steps are all mediated by HA, the activity noted here might be explained by the fact that MAC could prevent influenza virus or the viral genome from entering the host cells by interaction with the viral haemagglutinin protein. To ascertain whether the explanation was feasible, the interaction between MAC and the viral haemagglutinin was accessed by means of molecular dynamics (MD).

**Figure 5:**
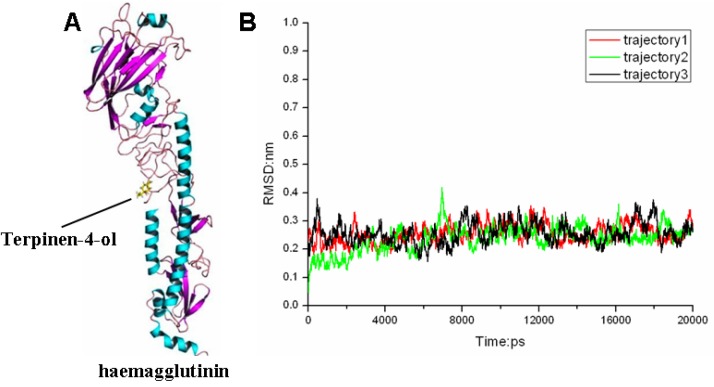
(**A**) The structures of complex obtained from docking calculations. (**B**) RMSDs of terpinen-4-ol-HA complex compared to their original conformations as a function of time.

MAC has been complete chemically defined and it was demonstrated that its antimicrobial activity could be principally attributed to terpinen-4-ol, the main active component [37,38,40,42,43,44]. Actually, terpinen-4-ol is the main bioactive antimicrobial component of essential oils derived from several aromatic plants [45,46,47]. On account of this, the interaction of terpinen-4-ol and the influenza virus haemagglutinin protein was predicted *in silico* to confirm the exact target and its active characteristics. From docking analyses, terpinen-4-ol was suggested to bind into a cavity near the fusion peptide (Figure 5). Figure 5A represents the initial structure of terpinen-4-ol-HA in the MD simulations. It is important to obtain a stable MD trajectory for subsequent analysis. Therefore, the root mean-square deviation (RMSD) values were used to measure the conformational stability of the terpinen-4-ol-HA complex during the MD simulations. From the RMSD curves in Figure 5B, it is suggested that the terpinen-4-ol-HA complexes obtained from MD simulations is relatively stable. There are three repeated RMSDs of the complex during the MD simulation and all of them are under 0.3 nm and the variations are within 0.1 nm.

**Figure 6:**
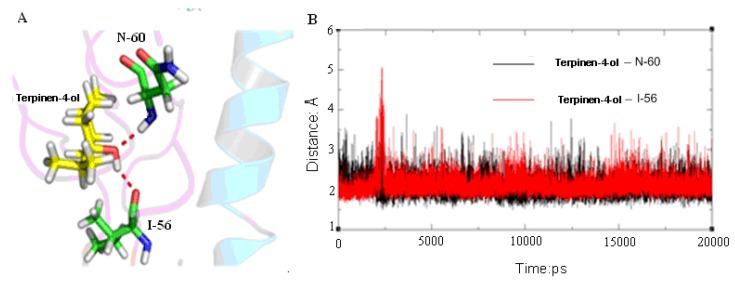
(**A**) Hydrogen bonds formed between terpinen-4-ol and residues in binding pocket. (**B**) The time dependent distance of terpinen-4-ol _I-56 (red) and terpinen-4-ol_N-60 (black).

As suggested by Russell [48], the fusion of virus and cell membranes is one of the key steps in the initial stages of infection. Comparison of the neutral-pH and fusion-pH structure indicates that at fusion-pH the membrane-distal domains of HA dissociate, and extensive structural reorganization occurs that involves extrusion of the “fusion peptide” from the interior of the neutral-pH structural. In its position in the fusion-pH structure [49], the fusion peptide is at the N terminus of a new 100-Å-long triple-helical coiled-coil, while the C-terminal membrane anchor is repositioned at the same end of the refolded molecule. Occupation of the membrane fusion site can stabilize the neutral-pH structure through inter- and intra-subunit interactions that presumably inhibit the conformational rearrangements required for membrane fusion. Therefore, we concluded that the terpinen-4-ol can stabilize the neutral-pH conformation of HA. From the MD simulation we can find terpinen-4-ol forms two strong hydrogen bonds with HA (Figure 6A). The time dependence of distances for these hydrogen bonds is shown in Figure 6B. It can be seen from Figure 6 that there are two firm hydrogen bonds residues of isoleucine (I)-56 and asparagine (N)-60 of HA2 averaging 2 Å between terpinen-4-ol and HA2, which play an important role in the stability of the complex. A hydrogen bond interaction was considered to form if the distance between the hydrogen donor and acceptor was less than 3.5 Å. We found that the hydrogen bonds between terpinen-4-ol and residues I-56 and N-60 of HA2 make significant contributions to the binding affinity. Therefore, we believe that the H-bond interaction between the hydroxyl moiety of terpinen-4-ol and I-56 and N-60 of HA2 stabilizes the terpinen-4-ol-HA complex in the MD simulation.

To explore the inhibition mechanisms of terpinen-4-ol with respect to its interaction with HA at the atomic level, the binding free energies were computed by means of the MM_GBSA. In particular, MM_GBSA combine molecular mechanics and continuum solvent models to estimate ligand binding affinities. The MM_GBSA calculation was constructed based a total of 250 snapshots that taken from the 15 ns to 20 ns. Importantly, the calculated binding free energy of the complex was −11.3647 kcal mol^−1^, which indicated that the terpinen-4-ol bonds strongly to HA protein. The results are listed in Table 1. The MD simulation based on the same initiating structure had been repeated for three times. For the complex, the electrostatic energy and the van der Waals energy favorably contributed to the binding free energies. The free energy of terpinen-4-ol binding to HA calculated by MM_GBSA method showed that the binding process is thermodynamically favorable. Therefore, we conclude that terpinen-4-ol binds to the membrane fusion site of HA and stabilizes the conformation of the fusion peptide through this interaction.

**Table 1:** Free Energy Calculations with MMGBSA method.

Contribution	Trajectory1			Trajectory2			Trajectory3	
	mean	std		mean	std		mean	std
E_elec_	−9.7112	2.3507		−9.4458	1.7139		−7.8287	3.4801
E_vdW_	−27.6956	2.0215		−27.3646	1.9431		−28.3145	2.0685
G_nonpolar_	−3.7310	0.0814		−3.7539	0.1006		−3.7499	0.1036
G_GB_	15.3067	1.9963		13.5294	1.2774		14.7480	1.7067
−TΔS	14.2991	5.4078		13.9034	4.9706		14.5704	5.1095
ΔG_GB_	−25.8310	1.9734		−27.0349	2.0078		−25.1450	2.6133
ΔG_GB(binding)_	−11.5319			−13.1215			−10.5746	

In conclusion, MD simulation was applied to clarify the three-dimensional structure of terpinen-4-ol bound to the active site. MD simulation revealed an optimal conformation of terpinen-4-ol-HA complex, in which the inhibitor forms two stable H-bonds with residues I-56 and N-60 of HA2 in the binding pocket. Moreover, MD simulation showed that this binding mode could stabilize the neutral pH conformation of HA. We believe that this property is important for antiviral activity of terpinen-4-ol. Understanding how terpinen-4-ol stabilized HA could provide a clue for the development of new influenza fusion inhibitors. The structural and mechanistic insights from the present study provide a valuable foundation for the structure-based design of more potent influenza fusion inhibitors.

## 3. Experimental

### 3.1. Bio-Safety

All experiments involving pathogenic influenza A viruses were performed in a bio-safety level 2 (BSL2) laboratory of Zhongshan School of Medicine of Sun Yat-sen University, Guangzhou, China.

### 3.2. Cells and Virus

Madin-Darby Canine Kidney (MDCK) cells maintained by our laboratory were grown in Dulbecco’s modified Eagle’s medium (DMEM, Invitrogen Corporation, New York, NY, USA) supplemented with 10% heat-inactivated fetal bovine serum (FBS, Thermo Scientific HyClone product line, Logan, UT, USA) at 37 °C, 5% CO_2_ (Heracell 150i, Thermo Scientific, Langenselbold, Germany). No antibiotics or anti-mycotic agents were used in cell or virus culture. 2009 H1N1 pandemic influenza virus strain, A/GuangzhouSB/01/2009(H1N1) (GZ01/09 for short) was a gift from the Guangdong Centers for Disease Control and Prevention that was propagated from clinical isolates and maintained in our laboratory. The virus strain was propagated in MDCK cells that were cultured in 0.02% TPCK- trypsin (Amresco Inc., Solon, OH, USA) at 37 °C, 5% CO_2_. The supernatant containing virus particles in MDCK cell culture was collected when 75%–100% CPE was observed. The virus was stored at −80 °C in aliquots until use.

### 3.3. Melaleuca Alternifolia Concentrate (MAC)

Hundred percent MAC (batch 270409) was provided by NeuMedix Biotechnology Pty Ltd, North Sydney, Australia. Preliminary experiments established the optimal solubility into dimethyl sulfoxide (DMSO) (Beijing Dingguo Changsheng Biotechnology Co. Ltd., Beijing, China) and the concentration of stock solution was 10% (v/v). For testing, the MAC stock solution was diluted by serum free DMEM media for working solutions with various concentrations.

### 3.4. Virus Titrations

The virus strain was titrated by standard Tissue Culture Infectious Dose_50_ (TCID_50_) Assay in MDCK cells. Briefly, MDCK cells were seeded in 96-well culture plates (about 5 × 10^4^ cell/well) in DMEM with 10% fetal bovine serum (FBS) for 12–24 h at 37 °C with 5% CO_2_. After cell propagation, growth medium was removed and 10 fold serial dilutions of the GZ01/09 virus suspension in DMEM media with 1 μg/mL TPCK-trypsin were added to the wells. The plate was incubated at 37 °C with 5% CO_2_, and morphological changes on the MDCK cells were observed microscopically every 12 h. The final CPE was recorded after 72 h. TCID50 was calculated by counting all the wells with 1–4 CPE as being positive. TCID50 was calculated by the Reed-Muench method [50].

### 3.5. MTT Assay to Determine the Cellular Viability of MDCK Cells

The cellular viability of MDCK cells was measured quantitatively by the reduction of formazan dye using MTT (3-(4,5-dimethylthiazol-2-yl)-2,5-diphenyltetrazolium bromide) (Beijing Dingguo Changsheng Biotechnology Co. Ltd., Beijing, China) assay. Briefly, confluent MDCK cell monolayer in 96-well culture plates was washed with sterile PBS and incubated for 3 h at 37 °C after 40 μL/well of MTT solution (5 mg/mL) was added into each well. When a purple precipitate was clearly visible, the liquid was carefully withdrawn without touching the sediment or the cells. DMSO at 100 μL/well was added to dissolve the purple formazan, and the absorbance at A490 nm was read with an Absorbance Microplate Reader (Gene Co. Ltd., Hong Kong, China).

### 3.6. Bioimaging in 96 Well Plates

The influence to entering host cell of the influenza virus by MAC treatment was determined by an immunofluorescence assay on MDCK cells in a 96 well plate. Briefly, MDCK cells were plated in a sterile 96-well plate about 10,000 cells/well. The influenza virus suspension treated with MAC of final concentration of 0.010% for 0.5 and 1 h at room temperature, virus suspension and maintain media for cell control were inoculated to the cell monolayer respectively, for 5 h in order for sufficient viral protein synthesis in the host cell. The cells were incubated at room temperature in the 3.7% formaldehyde 10 min for fixation; 0.1% Triton X-100 5 min for permeabilization and 3% fetal bovine serum 30 min for blocking. The influenza virus was stained with influenza A m1 (matrix protein 1) antibody (Santa Cruz Biotechnology, Inc. Santa Cruz, CA, USA) followed by Alexa Fluor^®^ 488 Goat Anti-Mouse IgG (H + L) (Molecular Probes, Invitrogen, Carlsbad, CA, USA). Finally, 50 µL per well of Fluoroshield™ with 4′,6-diamidino-2-phenylindole (DAPI, Sigma-Aldrich, Inc., St. Louis, MO, USA) was added and analyzed using an imaging instrument (Leica DMI4000B, Meyer Instruments, Inc., Houston, TX, USA).

### 3.7. Electron Microscopy Observation of the Influenza Virus Morphology

MDCK cells with or without treatment with MAC were observed under an electron microscope. The concentration and the treatment time of MAC were indicated in the figure legends. Each 10 μL of MAC-treated and untreated virus suspension was placed on a clean slide. Two copper grids were applied to float on the drops of virus suspensions using fine, clean forceps for 2 min. The bulk of the fluid was removed with the edge of the copper grid vertically on a strip of filter paper. Air dried the copper grid for 1 min. The copper grids were applied to float on a drop of 2% potassium phosphotungstate, using fine clean forceps, for 1 min. The bulk of the fluid was removed with the edge of the copper grid vertically on a strip of filter paper. Air dried the grid and examined in the electron microscope.

### 3.8. Statistical Analysis

The cell survival result in each group was expressed as the mean ± S.D. and the data was statistically compared with the relative control group using one-way analysis of variance (ANOVA), SPSS 17.0 for Windows software. *p* < 0.05 was considered to be statistically significant.

### 3.9. Molecular Docking

The structure of HA (PDB: 3AL4) [51] was used in the docking calculations. The program Autodock 4.0 [52] with a Lamarckian genetic algorithm is used to carry out the molecular docking. To evaluate the binding energies between the ligand and receptor, the AutoGrid program was used to generate the grid map with 80 × 80 × 80 points spaced equally at 0.375 Å is using. The number of GA runs is 200 and the energy evaluation is 25,000,000, other docking parameters were set to default values. At the end of the run, all docked conformations were clustered using a tolerance of 2 Å for root mean square deviations (RMSDs) and ranked based on docking energies.

### 3.10. Molecular Dynamics Simulations

The Amber 11.0 simulation suite [53] was used in molecular dynamics (MD) simulations and data analysis. An all-atom model of HA was generated using the tleap module on the basis of the initial model. To release conflicting contacts among residues, energy minimization was performed with steepest descent method for 500 steps, followed by conjugated gradient method for 500 steps. The protein was then solvated with water in a truncated tetrahedral periodic box (76.096 × 76.096 × 76.096 nm). The TIP3P [54] water model was used, and five Na^+^ counterions were added to neutralize the system. Prior to the production phase, the following equilibration protocol was applied. First, the solvent was relaxed by energy minimization while restraining the protein atomic positions with a harmonic potential. The system was then energy-minimized without restraints for 2,500 steps using a combination of steepest descent and conjugated gradient methods. The system was gradually heated from 0 to 300 K over 20 ps using the NVT enemble. Finally, 20-ns MD simulation was conducted at 1 atmosphere and 300 K with the NPT ensemble. During the simulation, the SHAKE [55] algorithm was applied to constrain the covalent bonds to hydrogen atoms. A time step of 2 fs and a non-bond interaction cutoff radius of 12.0 Å were used. Coordinates were saved every 1 ps during the entire process. The ff03 all atom Amber force field (AMBER ff03) developed by Duan *et al*. [56], which shows a good balance in the balance between helix and sheet, was used for the protein and the AMBER GAFF [57] was used for the ligand. The parameters for terpinen-4-ol were developed as follows: the electrostatic potential of terpinen-4-ol was obtained at the HF/6-31G basis set from GAUSSIAN 2003 [58] after a geometry optimization at the same level. The partial charges were derived by fitting the gas-phase electrostatic potential using the restrained electrostatic potential (RESP) method [59]. The missing interaction parameters in the ligand were generated using antechamber tools in Amber. The long-range electrostatic were calculated by the particle-mesh ewald (PME) method [60]. Then we used molecular mechanics generalized Born surface area (MM-GBSA) to estimated the binding energies at 192 AMD Opteron (tm) Processor CPUs (2.0 GHz) were used in the simulation process.

### 3.11. Binding Free Energy Calculation

The binding free energies (ΔG_bind_) were calculated using the MM-GBSA approach [61] inside the AMBER program. The first step of MM-GBSA method was the generation of multiple snapshots from an MD trajectory of the protein-ligand complex and a total 0f 50 snapshots were taken from the last 5 ns trajectory with an interval of 100 ps. For each snapshot, the free energy was calculated for each molecular species (complex, receptor, and ligand) using the following equation [62]:

ΔG_bind_ = G_com_ − G_rec_ − G_lig_ (1)

ΔG_bind_ = ΔE_mm_ + ΔG_solv_ - TΔS (2)

ΔG_mm_ = ΔE_elec_ + ΔE_vdw_ + ΔE_ini_ (3)

ΔG_solv_ = ΔG_GB_ + ΔG_np_ (4)

ΔG_np_ = γΔSASA + β (5)

where G_com_, G_rec_, and G_lig_ were the free energies for the complex, receptor, and ligand, respectively. ΔE_mm_ was the molecular mechanics energy of the molecule expressed as the sum of the internal energy of the molecule plus the electrostatics and van der Waals interactions; ΔG_solv_ was the solvation free energy of the molecule; T was the absolute temperature; and ΔS is entropy of the molecule. ΔE_elec_ was the Coulomb interaction, ΔE_vdw_ was the van der Waals interaction, and ΔE_ini_ was the sum of the bond, angle, and dihedral energies; in this case, ΔE_ini_ = 0. ΔG_GB_ is polar solvation contribution calculated by solving the GB equation [63] for MM_GBSA method. ΔG_np_ was the nonpolar solvation term; γ was the surface tension that was set to 0.0072 kal/(mol Å^2^);. ΔSASA is the solvent accessible surface area (Å^2^) that was estimated using the MOLSURF algorithm and β was a constant that was set to 0. The solvent probe radius was set to 1.4 Å to define the dielectric boundary around the molecular surface. The vibrational entropy contributions were estimated by NMODE analysis [64] and 50 snapshots were used in the NMODE analysis. To obtain the contribution of each the binding energy, MM_GBSA was used to decompose the interaction energies to each residue involved in the interaction but only considering molecular mechanics and solvation energies without the contribution of entropies.

## 4. Conclusions

In conclusion, we have proved that an herbal extract has significant effect against influenza virus. The cause of the effect was probably terpinen-4-ol, the main bioactive component, binding to the fusion peptide of the haemagglutinin protein on the surface of influenza virus. Because of the fusion peptide is high conservative, thus, the herbal extract, as the haemagglutinin conformational change inhibitors, *in vivo* studies are essential to confirm the *in vitro* data.
